# Dilithium disodium nickel(II) cyclo­hexa­phosphate dodeca­hydrate, Li_2_Na_2_NiP_6_O_18_·12H_2_O

**DOI:** 10.1107/S1600536812029960

**Published:** 2012-07-07

**Authors:** Sonia Abid, Salem S. Al-Deyab, Mohamed Rzaigui

**Affiliations:** a Laboratoire de Chimie des Matériaux, Faculté des Sciences de Bizerte, 7021 Zarzouna Bizerte, Tunisia; b Petrochemical Research Chair, College of Science, King Saud University, Riyadh, Saudi Arabia

## Abstract

The crystal structure of Li_2_Na_2_NiP_6_O_18_·12H_2_O is characterized by the presence of six-membered P_6_O_18_^6−^ phosphate ring anions (inter­nal symmetry -1) having a chair conformation and three different cations, *viz.* Li^+^, Na^+^ and Ni^2+^, to counterbalance the anionic charge. All atoms are in general positions except for nickel, which lies on a special position with site symmetry 2. Lithium has a tetra­hedral environment (LiO_4_), and sodium and nickel have octa­hedral environments [NaO_6_ and Ni(H_2_O)_6_, respectively]. The P_6_O_18_ rings are linked *via* corner sharing by NaO_6_ octa­hedra and LiO_4_ tetra­hedra to form a three-dimensional framework presenting tunnels running along [010] in which the six-coordinated Ni^2+^ cations are located. The structure is stabilized by a network of O—H⋯O hydrogen bonds.

## Related literature

For the crystal chemistry of cyclic phosphates, see: Averbuch-Pouchot & Durif (1996 ▶). For related structures containing cyclo­hexa­phosphate rings, see: Abid *et al.* (2011 ▶); Amri *et al.* (2009 ▶); Marouani *et al.* (2010 ▶). For hydrogen bonding, see: Blessing (1986 ▶). For the synthesis, see: Schülke & Kayser (1985 ▶).

## Experimental

### 

#### Crystal data

Li_2_Na_2_NiP_6_O_18_·12H_2_O
*M* *_r_* = 808.58
Monoclinic, 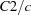

*a* = 17.728 (9) Å
*b* = 10.213 (2) Å
*c* = 14.801 (7) Å
β = 112.04 (4)°
*V* = 2484.0 (18) Å^3^
*Z* = 4
Ag *K*α radiation
λ = 0.56085 Å
μ = 0.69 mm^−1^
*T* = 298 K
0.40 × 0.35 × 0.30 mm

#### Data collection

Nonius MACH-3 diffractometer
Absorption correction: part of the refinement model (Δ*F*) (Walker & Stuart, 1983 ▶) *T*_min_ = 0.769, *T*_max_ = 0.819
7168 measured reflections
6076 independent reflections
4296 reflections with *I* > 2σ(*I*)
*R*_int_ = 0.035
2 standard reflections every 120 min intensity decay: 2%

#### Refinement

*R*[*F* ^2^ > 2σ(*F* ^2^)] = 0.044
*wR*(*F* ^2^) = 0.102
*S* = 1.07
6076 reflections
186 parameters
2 restraints
H-atom parameters constrained
Δρ_max_ = 0.80 e Å^−3^
Δρ_min_ = −0.61 e Å^−3^

### 

Data collection: *CAD-4 EXPRESS* (Enraf–Nonius, 1994 ▶); cell refinement: *CAD-4 EXPRESS*; data reduction: *XCAD4* (Harms & Wocadlo, 1995 ▶); program(s) used to solve structure: *SHELXS86* (Sheldrick, 2008 ▶); program(s) used to refine structure: *SHELXL97* (Sheldrick, 2008 ▶); molecular graphics: *ORTEP-3 for Windows* (Farrugia, 1997 ▶); software used to prepare material for publication: *WinGX* (Farrugia, 1999 ▶).

## Supplementary Material

Crystal structure: contains datablock(s) I, global. DOI: 10.1107/S1600536812029960/ff2073sup1.cif

Structure factors: contains datablock(s) I. DOI: 10.1107/S1600536812029960/ff2073Isup2.hkl

Additional supplementary materials:  crystallographic information; 3D view; checkCIF report

## Figures and Tables

**Table 1:** Selected bond lengths (Å)

Na1—O1^i^	2.4205 (19)
Na1—O5^ii^	2.384 (2)
Na1—O7^iii^	2.3737 (19)
Na1—O10^iii^	2.556 (2)
Na1—O11	2.546 (2)
Na1—O12	2.323 (2)
Li—O2^i^	1.927 (4)
Li—O8	1.930 (5)
Li—O10	1.964 (5)
Li—O11	1.972 (5)
Ni1—O13	2.0469 (16)
Ni1—O15	2.0572 (15)
Ni1—O14	2.0693 (18)

Symmetry codes: (i) 
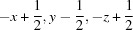
; (ii) 
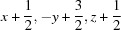
; (iii) 
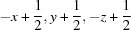
.

**Table 2:** Hydrogen-bond geometry (Å, °)

*D*—H⋯*A*	*D*—H	H⋯*A*	*D*⋯*A*	*D*—H⋯*A*
O10—H110⋯O15^iii^	0.85	1.96	2.795 (3)	165
O10—H210⋯O4^iv^	0.87	2.03	2.851 (3)	159
O11—H111⋯O14^iii^	0.82	2.01	2.811 (3)	164
O11—H211⋯O2	0.86	2.25	3.031 (3)	150
O12—H112⋯O12^v^	0.86	2.44	3.058 (4)	130
O12—H212⋯O3^vi^	0.86	2.48	3.304 (3)	161
O12—H212⋯O4^vi^	0.86	2.52	3.166 (3)	133
O15—H115⋯O4^vii^	0.88	1.87	2.741 (3)	171
O15—H215⋯O5^i^	0.83	1.85	2.673 (3)	174
O14—H114⋯O8	0.85	1.94	2.758 (3)	163
O14—H214⋯O7^viii^	0.86	1.78	2.643 (3)	174
O13—H113⋯O2^i^	0.83	1.97	2.789 (3)	167
O13—H213⋯O1^ii^	0.84	1.84	2.677 (3)	174

Symmetry codes: (i) 
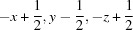
; (ii) 
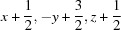
; (iii) 
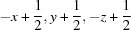
; (iv) 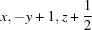; (v) 
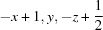
; (vi) 
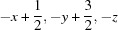
; (vii) 
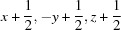
; (viii) 
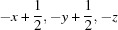
.

**Fig. 1.**
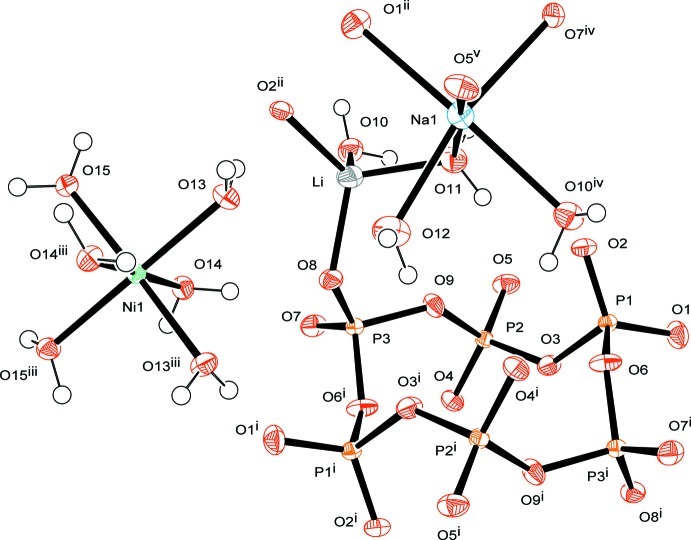
ORTEP-3 (Farrugia, 1997) view of (I) with atom numbering scheme. Displacement ellipsoids for non-H atoms are drawn at the 50% probability level. [Symmetry codes: (i) 0.5-x, 1.5-y, -z; (ii) 0.5-x, -0.5+y, 0.5-z; (iii) 1-x, y, 0.5-z; (iv) 0.5-x, 0.5+y, 0.5-z; (v) 0.5+x, 1.5-y, 0.5+z]

**Fig. 2.**
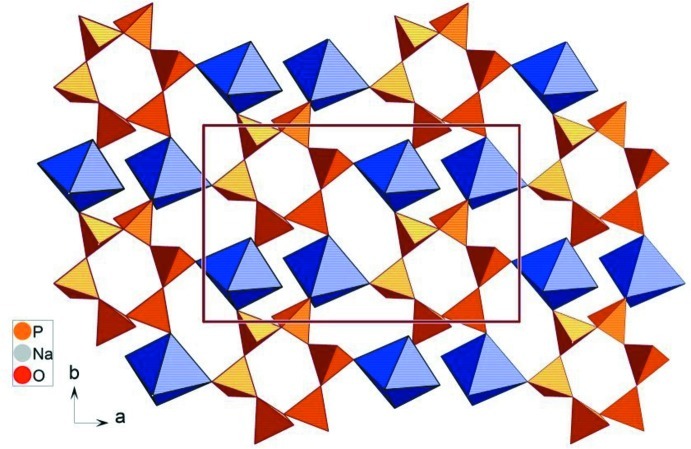
View of [Na2(P6O18)]n4n- developed along the c axis.

**Fig. 3.**
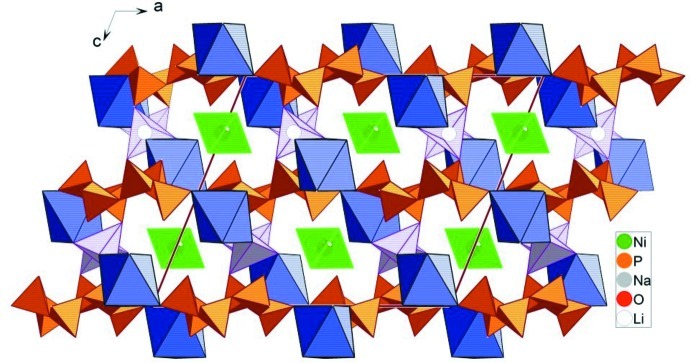
Projection of the structure of Li2Na2NiP6O18.12H2O along the b axis

## supplementary crystallographic information

### Comment

Cyclophosphohates, corresponding to the anionic formula [P_n_O_3n_]^n-^, constitute the second important family of condensed phosphates after the polyphosphates. The identified cyclic anions, built by n corner-sharing PO_4_ tetrahedra, correspond to n = 3, 4, 5, 6, 8, 9, 10 and 12. The phosphoric ring anion corresponding to n = 6, called cyclohexaphosphate, has been associated to numerous organic and/or inorganic cations (Averbuch-Pouchot & Durif, 1996). But its association to three mixed cations is still very limited. In this work, we report the preparation and the structural investigation of a novel dilithium disodium nickel cyclohexaphosphate dodecahydrate, Li_2_Na_2_NiP_6_O_18_.12H_2_O (I). To our knowledge, there is no cyclohexaphosphate with a mixture of two alkalines and bivalent cations. The partial three-dimensional plot in Fig.1 illustrates the connection ion-oxygen polyhedra and the phosphoric ring in the crystal structure of the title compound. Among the 21 atoms included in the asymmetric unit of this structure, only the Ni atom is in a special position ((Wyckoff position 4 e, site symmetry 2)). The Li, Na and Ni atoms are coordinated to four, for the first one, and to six, for the last two, oxygen atoms. The NaO_6_ and P_6_O_18_ entities are linked in an alternating manner to generate a two-dimensional open framework, forming so layers parallel to the (a,b) plane (Fig. 2). Adjacent layers are connected by the LiO_4_ tetrahedra to generate a three dimensional structure exhibiting channels running along the *b* axis (Fig. 3). Inside these channels, the Ni^2+^ cation is coordinated by six water molecules. The [Ni(H_2_O)_6_]^2+^ octahedron is almost regular with Ni–O distances ranging from 2.0462 (16) to 2.0691 (18) Å. The smallest distance between two octahedral centers is 9.069 Å. The cyclic anion (P_6_O_18_)^6-^ has a chair conformation with geometrical characteristics that show no significant difference deviation from those observed in other cyclohexaphosphates having the same internal symmetry -1 (Abid *et al.* 2011,Amri *et al.*2009; Marouani *et al.*2010). In addition to its interactions with the metallic cations, the phosphoric anion establish with the water molecules an important hydrogen-bonding scheme. The examination of this latter shows the existence of strong hydrogen bonds with distances O···O ranging from 2.643 (3) to 2.677 (3) Å and other weaker ones, with O···O distances falling from 2.741 (3) to 3.304 (3) Å (Blessing, 1986).

### Experimental

Li_2_Na_2_NiP_6_O_18_.12H_2_O was prepared by mixing Li_6_P_6_O_18_.6H_2_O (0.5 g, 5 mmol), NiCl_2_.6H_2_O (0.71 g, 3 mmol), and NaNO_3_ (0.03 g, 0.4 mmol) in 50 ml of distillated water and stirring for 30 min at temperature room. The obtained solution was allowed to stand in air until formation of good greenish single crystals of the title compound. Its chemical formula was determined by X-ray diffraction. The used Li_6_P_6_O_18_.6H_2_O was prepared according to the procedure of Schülke and Kayser (Schülke & Kayser, 1985)

### Refinement

Hydrogen atoms were placed in geometrically idealized positions (O—H =0.85 Å) and treated as riding with *U*_iso_(H) = 1.2 *U*_eq_ of their parent atoms.

### Crystal data

**Table:**

Li_2_Na_2_NiP_6_O_18_·12H_2_O	*F*(000) = 1640
*M**_r_* = 808.58	*D*_x_ = 2.162 Mg m^−^^3^
Monoclinic, *C*2/*c*	Ag *K*α radiation, λ = 0.56085 Å
Hall symbol: -C 2yc	Cell parameters from 25 reflections
*a* = 17.728 (9) Å	θ = 8.3–10.8°
*b* = 10.213 (2) Å	µ = 0.69 mm^−^^1^
*c* = 14.801 (7) Å	*T* = 298 K
β = 112.04 (4)°	Prism, green
*V* = 2484.0 (18) Å^3^	0.40 × 0.35 × 0.30 mm
*Z* = 4	

### Data collection

**Table:**

Nonius MACH-3 diffractometer	4296 reflections with *I* > 2σ(*I*)
Radiation source: fine-focus sealed tube	*R*_int_ = 0.035
Graphite monochromator	θ_max_ = 28.0°, θ_min_ = 2.3°
non–profiled ω scans	*h* = −29→27
Absorption correction: part of the refinement model (Δ*F*) (Walker & Stuart, 1983)	*k* = −1→17
*T*_min_ = 0.769, *T*_max_ = 0.819	*l* = −1→24
7168 measured reflections	2 standard reflections every 120 min
6076 independent reflections	intensity decay: 2%

### Refinement

**Table:**

Refinement on *F*^2^	Primary atom site location: structure-invariant direct methods
Least-squares matrix: full	Secondary atom site location: difference Fourier map
*R*[*F*^2^ > 2σ(*F*^2^)] = 0.044	Hydrogen site location: inferred from neighbouring sites
*wR*(*F*^2^) = 0.102	H-atom parameters constrained
*S* = 1.07	*w* = 1/[σ^2^(*F*_o_^2^) + (0.0389*P*)^2^ + 4.028*P*] where *P* = (*F*_o_^2^ + 2*F*_c_^2^)/3
6076 reflections	(Δ/σ)_max_ = 0.003
186 parameters	Δρ_max_ = 0.80 e Å^−^^3^
2 restraints	Δρ_min_ = −0.61 e Å^−^^3^

### Special details

**Table:**

Geometry. All e.s.d.'s (except the e.s.d. in the dihedral angle between two l.s. planes) are estimated using the full covariance matrix. The cell e.s.d.'s are taken into account individually in the estimation of e.s.d.'s in distances, angles and torsion angles; correlations between e.s.d.'s in cell parameters are only used when they are defined by crystal symmetry. An approximate (isotropic) treatment of cell e.s.d.'s is used for estimating e.s.d.'s involving l.s. planes.
Refinement. Refinement of *F*^2^ against ALL reflections. The weighted *R*-factor *wR* and goodness of fit *S* are based on *F*^2^, conventional *R*-factors *R* are based on *F*, with *F* set to zero for negative *F*^2^. The threshold expression of *F*^2^ > σ(*F*^2^) is used only for calculating *R*-factors(gt) *etc*. and is not relevant to the choice of reflections for refinement. *R*-factors based on *F*^2^ are statistically about twice as large as those based on *F*, and *R*- factors based on ALL data will be even larger.

### Fractional atomic coordinates and isotropic or equivalent isotropic displacement parameters (Å^2^)

**Table:**

	*x*	*y*	*z*	*U*_iso_*/*U*_eq_	
Na1	0.38942 (6)	0.74638 (9)	0.40089 (7)	0.02302 (18)	
Li	0.2602 (3)	0.4883 (4)	0.2527 (3)	0.0258 (8)	
O8	0.28521 (9)	0.47433 (15)	0.13696 (11)	0.0182 (3)	
O9	0.16879 (9)	0.61570 (15)	0.04655 (11)	0.0209 (3)	
O10	0.14882 (10)	0.42811 (17)	0.22717 (13)	0.0249 (3)	
H110	0.1134	0.4875	0.2021	0.030*	
H210	0.1389	0.4015	0.2772	0.030*	
O11	0.25138 (10)	0.67633 (16)	0.27740 (13)	0.0260 (3)	
H111	0.2134	0.6824	0.2962	0.031*	
H211	0.2414	0.7291	0.2287	0.031*	
O12	0.42660 (15)	0.6531 (2)	0.28051 (17)	0.0437 (5)	
H112	0.4775	0.6725	0.3012	0.052*	
H212	0.4065	0.6762	0.2205	0.052*	
P1	0.15702 (3)	0.94221 (5)	0.03614 (4)	0.01251 (9)	
P3	0.21812 (3)	0.48781 (5)	0.04013 (4)	0.01248 (9)	
P2	0.09260 (3)	0.68397 (5)	−0.03712 (4)	0.01298 (9)	
O4	0.07807 (9)	0.62089 (15)	−0.13208 (11)	0.0196 (3)	
O2	0.16821 (9)	0.89346 (15)	0.13535 (10)	0.0177 (3)	
O7	0.16162 (10)	0.37702 (15)	0.00214 (12)	0.0216 (3)	
O5	0.02574 (9)	0.69398 (17)	−0.00121 (12)	0.0227 (3)	
O6	0.24650 (8)	0.97174 (17)	0.04034 (11)	0.0208 (3)	
O1	0.10493 (10)	1.05743 (15)	−0.00194 (12)	0.0243 (3)	
O3	0.12913 (10)	0.82657 (15)	−0.04140 (11)	0.0216 (3)	
Ni1	0.5000	0.24488 (3)	0.2500	0.01249 (7)	
O15	0.48362 (9)	0.09969 (14)	0.33724 (11)	0.0172 (3)	
H115	0.5184	0.0349	0.3502	0.021*	
H215	0.4838	0.1271	0.3902	0.021*	
O14	0.37693 (8)	0.24893 (14)	0.16516 (11)	0.0172 (3)	
H114	0.3569	0.3256	0.1540	0.021*	
H214	0.3616	0.2121	0.1087	0.021*	
O13	0.47785 (9)	0.38693 (15)	0.33445 (11)	0.0197 (3)	
H113	0.4355	0.3760	0.3454	0.024*	
H213	0.5162	0.4089	0.3860	0.024*	

### Atomic displacement parameters (Å^2^)

**Table:**

	*U*^11^	*U*^22^	*U*^33^	*U*^12^	*U*^13^	*U*^23^
Na1	0.0231 (4)	0.0223 (4)	0.0243 (4)	−0.0004 (3)	0.0097 (3)	−0.0018 (4)
Li	0.0293 (19)	0.029 (2)	0.0183 (18)	0.0059 (17)	0.0085 (15)	0.0022 (16)
O8	0.0169 (6)	0.0231 (7)	0.0128 (6)	0.0045 (5)	0.0036 (5)	0.0027 (5)
O9	0.0216 (6)	0.0225 (7)	0.0143 (6)	0.0095 (5)	0.0019 (5)	−0.0022 (6)
O10	0.0241 (7)	0.0267 (8)	0.0248 (8)	0.0044 (6)	0.0104 (6)	0.0044 (7)
O11	0.0289 (8)	0.0247 (7)	0.0284 (9)	−0.0001 (6)	0.0155 (7)	0.0001 (7)
O12	0.0520 (13)	0.0494 (12)	0.0371 (11)	0.0085 (10)	0.0251 (10)	0.0033 (10)
P1	0.01273 (18)	0.01321 (18)	0.0114 (2)	−0.00086 (15)	0.00431 (15)	0.00013 (16)
P3	0.01266 (18)	0.01344 (19)	0.0114 (2)	−0.00034 (15)	0.00457 (15)	0.00006 (16)
P2	0.01426 (19)	0.01291 (18)	0.0112 (2)	−0.00007 (15)	0.00409 (16)	−0.00088 (16)
O4	0.0237 (7)	0.0195 (6)	0.0145 (6)	−0.0021 (5)	0.0059 (5)	−0.0057 (5)
O2	0.0196 (6)	0.0219 (6)	0.0131 (6)	−0.0040 (5)	0.0078 (5)	0.0001 (5)
O7	0.0271 (7)	0.0190 (6)	0.0192 (7)	−0.0097 (6)	0.0094 (6)	−0.0030 (6)
O5	0.0182 (6)	0.0324 (8)	0.0196 (7)	0.0028 (6)	0.0096 (6)	0.0047 (6)
O6	0.0143 (5)	0.0354 (8)	0.0134 (6)	−0.0041 (6)	0.0061 (5)	0.0029 (6)
O1	0.0257 (7)	0.0206 (7)	0.0226 (8)	0.0085 (6)	0.0045 (6)	0.0000 (6)
O3	0.0344 (8)	0.0162 (6)	0.0148 (6)	−0.0090 (6)	0.0100 (6)	−0.0035 (5)
Ni1	0.01256 (13)	0.01290 (14)	0.01169 (14)	0.000	0.00419 (11)	0.000
O15	0.0206 (6)	0.0162 (6)	0.0149 (6)	0.0014 (5)	0.0069 (5)	0.0012 (5)
O14	0.0160 (5)	0.0176 (6)	0.0159 (6)	0.0017 (5)	0.0035 (5)	−0.0010 (5)
O13	0.0193 (6)	0.0220 (7)	0.0186 (7)	−0.0005 (5)	0.0080 (5)	−0.0051 (6)

### Geometric parameters (Å, º)

**Table:**

Na1—O1^i^	2.4205 (19)	P3—O7	1.4754 (16)
Na1—O5^ii^	2.384 (2)	P3—O6^iv^	1.5948 (17)
Na1—O7^iii^	2.3737 (19)	P2—O5	1.4735 (17)
Na1—O10^iii^	2.556 (2)	P2—O4	1.4782 (17)
Na1—O11	2.546 (2)	P2—O3	1.6047 (16)
Na1—O12	2.323 (2)	O2—Li^iii^	1.927 (4)
Li—O2^i^	1.927 (4)	O7—Na1^i^	2.3737 (19)
Li—O8	1.930 (5)	O5—Na1^v^	2.384 (2)
Li—O10	1.964 (5)	O6—P3^iv^	1.5948 (17)
Li—O11	1.972 (5)	O1—Na1^iii^	2.4205 (19)
O8—P3	1.4860 (17)	Ni1—O13	2.0469 (16)
O9—P3	1.5944 (16)	Ni1—O13^vi^	2.0469 (16)
O9—P2	1.6088 (17)	Ni1—O15^vi^	2.0572 (15)
P1—O1	1.4712 (16)	Ni1—O15	2.0572 (15)
P1—O2	1.4917 (17)	Ni1—O14^vi^	2.0693 (18)
P1—O3	1.5908 (16)	Ni1—O14	2.0693 (18)
P1—O6	1.5929 (17)		
			
O12—Na1—O7^iii^	167.54 (8)	O7—P3—O9	110.00 (10)
O12—Na1—O5^ii^	93.23 (9)	O8—P3—O9	106.07 (9)
O7^iii^—Na1—O5^ii^	91.05 (7)	O7—P3—O6^iv^	108.36 (9)
O12—Na1—O1^i^	100.91 (8)	O8—P3—O6^iv^	110.34 (9)
O7^iii^—Na1—O1^i^	90.64 (7)	O9—P3—O6^iv^	102.15 (9)
O5^ii^—Na1—O1^i^	91.76 (7)	O5—P2—O4	119.77 (10)
O12—Na1—O11	78.89 (9)	O5—P2—O3	110.02 (10)
O7^iii^—Na1—O11	96.33 (7)	O4—P2—O3	106.67 (9)
O5^ii^—Na1—O11	171.92 (7)	O5—P2—O9	108.00 (10)
O1^i^—Na1—O11	91.45 (7)	O4—P2—O9	109.84 (9)
O12—Na1—O10^iii^	78.55 (8)	O3—P2—O9	100.90 (9)
O7^iii^—Na1—O10^iii^	89.14 (7)	P1—O2—Li^iii^	118.66 (16)
O5^ii^—Na1—O10^iii^	101.04 (7)	P3—O7—Na1^i^	123.98 (10)
O1^i^—Na1—O10^iii^	167.20 (7)	P2—O5—Na1^v^	124.59 (10)
O11—Na1—O10^iii^	75.86 (7)	P1—O6—P3^iv^	133.24 (10)
O2^i^—Li—O8	115.4 (2)	P1—O1—Na1^iii^	121.77 (10)
O2^i^—Li—O10	107.4 (2)	P1—O3—P2	131.77 (10)
O8—Li—O10	110.8 (2)	O13—Ni1—O13^vi^	89.73 (9)
O2^i^—Li—O11	113.7 (2)	O13—Ni1—O15^vi^	177.22 (6)
O8—Li—O11	107.3 (2)	O13^vi^—Ni1—O15^vi^	91.32 (7)
O10—Li—O11	101.3 (2)	O13—Ni1—O15	91.32 (7)
P3—O8—Li	118.83 (15)	O13^vi^—Ni1—O15	177.22 (6)
P3—O9—P2	129.04 (10)	O15^vi^—Ni1—O15	87.76 (9)
Li—O10—Na1^i^	109.74 (15)	O13—Ni1—O14^vi^	90.89 (7)
Li—O11—Na1	106.55 (15)	O13^vi^—Ni1—O14^vi^	87.49 (7)
O1—P1—O2	118.46 (10)	O15^vi^—Ni1—O14^vi^	91.73 (6)
O1—P1—O3	109.69 (10)	O15—Ni1—O14^vi^	89.92 (6)
O2—P1—O3	110.66 (9)	O13—Ni1—O14	87.49 (7)
O1—P1—O6	109.65 (10)	O13^vi^—Ni1—O14	90.89 (7)
O2—P1—O6	105.21 (9)	O15^vi^—Ni1—O14	89.92 (6)
O3—P1—O6	101.78 (9)	O15—Ni1—O14	91.73 (6)
O7—P3—O8	118.66 (10)	O14^vi^—Ni1—O14	177.71 (8)

Symmetry codes: (i) −*x*+1/2, *y*−1/2, −*z*+1/2; (ii) *x*+1/2, −*y*+3/2, *z*+1/2; (iii) −*x*+1/2, *y*+1/2, −*z*+1/2; (iv) −*x*+1/2, −*y*+3/2, −*z*; (v) *x*−1/2, −*y*+3/2, *z*−1/2; (vi) −*x*+1, *y*, −*z*+1/2.

### Hydrogen-bond geometry (Å, º)

**Table:**

*D*—H···*A*	*D*—H	H···*A*	*D*···*A*	*D*—H···*A*
O10—H110···O15^iii^	0.85	1.96	2.795 (3)	165
O10—H210···O4^vii^	0.87	2.03	2.851 (3)	159
O11—H111···O14^iii^	0.82	2.01	2.811 (3)	164
O11—H211···O2	0.86	2.25	3.031 (3)	150
O12—H112···O12^vi^	0.86	2.44	3.058 (4)	130
O12—H212···O3^iv^	0.86	2.48	3.304 (3)	161
O12—H212···O4^iv^	0.86	2.52	3.166 (3)	133
O15—H115···O4^viii^	0.88	1.87	2.741 (3)	171
O15—H215···O5^i^	0.83	1.85	2.673 (3)	174
O14—H114···O8	0.85	1.94	2.758 (3)	163
O14—H214···O7^ix^	0.86	1.78	2.643 (3)	174
O13—H113···O2^i^	0.83	1.97	2.789 (3)	167
O13—H213···O1^ii^	0.84	1.84	2.677 (3)	174

Symmetry codes: (i) −*x*+1/2, *y*−1/2, −*z*+1/2; (ii) *x*+1/2, −*y*+3/2, *z*+1/2; (iii) −*x*+1/2, *y*+1/2, −*z*+1/2; (iv) −*x*+1/2, −*y*+3/2, −*z*; (vi) −*x*+1, *y*, −*z*+1/2; (vii) *x*, −*y*+1, *z*+1/2; (viii) *x*+1/2, −*y*+1/2, *z*+1/2; (ix) −*x*+1/2, −*y*+1/2, −*z*.
